# Medical Education in the United States

**Published:** 1918-11

**Authors:** 


### MEDICAL EDUCATION IN THE UNITED STATES

We are publishing this week, for the eighteenth consecutive year, statistics regarding medical education in the United States. The great improvements that have been made during these eighteen years have been set forth in previous reports and are generally known to most of our readers. A study of the statistics will be well worth while, however, and, for those who may not have seen the previous reports, a few statements will be of interest.

Following the Civil War, the number of medical schools rapidly increased until in 1900 there were 160 medical schools in the United States—more than in all the rest of the world. Most of the schools were dependent on the student's fees for maintenance, and some were making profits from that source. Medical teaching was largely overshadowed by the energy expended to secure large enrollments. It was a common practice to issue pretentious announcements and extravagantly worded circulars; and even to send out solicitors who were paid commissions for such students as should be enrolled. Number, not quality, was sought; few students were so grossly ignorant that they could not gain admission. In fact, statistics show that in 1904 only four medical colleges were requiring any college work for admission, and only from 20 to 25 per cent. were actually requiring a four-year high school education. Under the methods pursued, it is not surprising that in 1904 the number of medical students reached the amazing total of 28,142, and that in that year there were graduated 5,747 physicians; nor is it a wonder that at the present time the Surgeon-General has to reject so many applicants for the Medical Reserve Corps because of their lack of professional training.

The campaign of publicity started by *The Journal* in 1900 led, in 1904, to the creation of the Council on Medical Education, which was placed in charge of the campaign for improvement. Minimal and ideal standards of education were drawn up; the merger of from two to five medical schools into one in each of various cities and states was advocated; annual conferences for the discussion of educational problems were held; the need of additional endowments for medical education was shown, and finally, all medical schools were inspected and classified. A wide publicity in the columns of *The Journal* and in reports and pamphlets was given to the conditions found and to the Council's classifications, and improvements since then have been rapid. At present, largely through mergers, the number of colleges has been reduced to ninety; the number of students has been reduced to 13,630, and the number of graduates to 2,670.[1] These lower figures represent the normal decrease that was expected under the increased entrance requirements, and are not due to the war. In Journal A. M. A., Aug. 17, p. 542, the enrollments in the first three classes show an increase over previous years. Next year, therefore, unless the war makes an unexpected demand on the medical student body, the total enrollment in all medical schools and the total number of graduates should show an increase over the present year.

While in the totals of all colleges, students and graduates there has been a decrease, on the other hand, as shown in the accompanying tabulation, there has been a decided increase in the number of colleges that have enforced higher entrance requirements and in the numbers of students and graduates who possessed the higher entrance qualifications.

---

[1] Altogether 2,807 students successfully completed the courses of the senior year. From 137, however, in the Universities of California and Minnesota and in Rush Medical College, the degrees have been withheld pending the completion of a hospital internship.

| Entrance Requirements. | Colleges 1904 | | 1918 | |
|---|---|---|---|---|
| | No. | % | No. | % |
| Four-yr. high school education or less* | 158 | 97.5 | 7 | 7.8 |
| One year of college work | .... | .... | 34 | 37.8 |
| Two years of college work | 4 | 2.5 | 49 | 54.4 |
| Totals | 162 | .... | 90 | .... |

| Entrance Requirements. | Students 1904 | | 1918 | |
|---|---|---|---|---|
| | No. | % | No. | % |
| Four-yr. high school education or less* | 26,391 | 93.8 | 631 | 4.6 |
| One year of college work | .... | .... | 5,944 | 43.6 |
| Two years of college work | 1,761 | 6.2 | 7,055 | 51.8 |
| Totals | 28,142 | .... | 13,630 | .... |

| Entrance Requirements. | Graduates 1904 | | 1918 | |
|---|---|---|---|---|
| | No. | % | No. | % |
| Four-yr. high school education or less* | 5,378 | 93.6 | 258 | 9.7 |
| One year of college work | .... | .... | 1,147 | 43.0 |
| Two years of college work | 369 | 6.4 | 1,265 | 47.3 |
| Totals | 5,747 | .... | 2,670[1] | .... |

Instead of four (2.5 per cent.) medical schools which in 1904 required any college work for admission, now eighty-three (92.2 per cent.) are requiring one or two years of such work; instead of only 1,761 (6.2 per cent.) students enrolled in the higher standard colleges in 1904, during the year 12,999 (95.3 per cent.) students were enrolled in the higher standard colleges, and instead of only 369 (6.4 per cent.) graduates who were turned out by the higher standard colleges in 1904, at the end of the last session, 2,412 (90.3 per cent.) graduated from those institutions. In addition to the improvement in preliminary qualifications, which represents only one of the improvements brought about in medical education, it should also be stated that greatly increased endowments have been secured, many schools having received hundreds of thousands of dollars, while a score or more have received

* It is not probable that in 1904 more than about thirty colleges (20 per cent.) were actually requiring a four-year high school education as a minimum for admission.

gifts of millions; most of the medical schools have erected new buildings, have established better equipped laboratories, have obtained more abundant clinical facilities, and, still more important, have employed larger numbers of skilled full-time teachers and developed better methods of teaching in both the laboratory and the clinical portions of the school. Instead of the large proportion of lectures or lecture clinics that constituted so great a part of the former curriculum, now the student gets most of his clinical training at the bedside of the patient, in small group clinics or in having patients individually assigned to him.

Since the proportion of physicians to population in the United States is still one to every 739 people, as compared with one to every 1,500 to 2,500 in the European countries, it is evident that the reduction in the output of physicians each year has not been serious. On the contrary, there has been a large increase in the number of those who are much better qualified, both by preliminary education and medical training, to care adequately for the sick, and to take a more active part in the prevention of disease and the promotion of public health.—Reprint Editorial, *Journal A. M. A.*, Aug. 17, 1918.

